# Supplementary material for: Supervised pulmonary tele-rehabilitation and individualized home-based pulmonary rehabilitation for patients with COPD, unable to participate in center-based programs. The protocol for a multicenter randomized controlled trial - the REPORT study
Source: PLoS One. 2025 Jan 7;20(1):e0312742. doi: 10.1371/journal.pone.0312742 (PMC11706455; doi:10.1371/journal.pone.0312742)
Supplement: S1 Protocol — (PDF) [file pone.0312742.s001.pdf]

# 1. Rethink Pulmonary Rehabilitation – the REPORT Study

## 2. Rationale

The common disease chronic obstructive pulmonary disease (COPD) is closely associated with smoking, and costs DKK 8.5 billion annually in citizens´ contact and treatment within the health care system[1]. It is estimated that 12% of all adult Danes above the age of 45 years have COPD[2]. Improved treatment options and increasing life expectancy imply that the number of people with rehabilitation-requiring COPD increases and places increased demands on best possible use of the limited resources in the health care system, not least in the years to come[3].

Pulmonary rehabilitation (PR) is one of the cornerstones of care for people with COPD together with smoking cessation and medical treatment. There is robust evidence that pulmonary rehabilitation improves exercise capacity, enhances health-related quality of life (QoL) and reduces healthcare utilization[4–6]. PR is strongly recommended in guidelines for COPD management[5,7]. More than 85% of European countries have implemented an outpatient model, including Denmark, where participants attend two to three sessions each week of supervised exercise and self-management training for a period of eight weeks or more[8]. Despite the compelling evidence for its benefits, pulmonary rehabilitation is delivered to less than 30% of patients with COPD who would benefit from the intervention[9–13]. Referral to PR and access to PR are particularly challenging, and especially for those with the most progressed stages of the disease[12,14,15].

We recently completed a randomized clinical trial showing that approximately 1,100 patients annually are offered conventional hospital- and community-based PR during routine consultations in the Capital Region of Denmark, but of these at least 700 patients decline participation[14,16]. Frequent transport to the center-based program, in the setting of distressing dyspnea and mobility limitation, is regularly reported as an access-barrier to attendance in hospitals and community health-care centers and yet the delivery of PR has not changed significantly in 30 years[9–11,17,18].

Much is unknown regarding COPD patients unable to participate in conventional PR programs as no studies have been conducted to specifically intervene in this group. Important knowledge in terms of symptoms (respiratory, anxiety, depression, impaired QoL, body pain and sleep quality) and physical functioning (exercise capacity and objectively measured physical activity), and response to alternative rehabilitation models is a black box with no substantial research, except for studies on qualitative perceived enablers and barriers[10,11,17]. There is a key gap in evidence for new rehabilitation models. The effectiveness and feasibility of home-based pulmonary rehabilitation (HPR) and pulmonary tele-rehabilitation (PTR) have never been investigated in this specific group "conventional none-participant" of patients with COPD, but it is strongly endorsed as an essential research scope by the American Thoracic Society/European Respiratory Society[9].

Home-based pulmonary rehabilitation[19] and PTR[14] are two alternative models using health-care supportive technology that have proven equivalent or non-inferiority to the conventional PR programs in patients with COPD who are able and willing to participate in conventional PR[14,19,20].

However, for HPR and PTR to fulfil their promises, they must be accessible to the target groups, be well accepted by users, deliver the essential components of PR, and be easy to implement. Furthermore, the results after the interventions must be of clinical relevance, i.e. beneficial and superior to the current 'usual care' (medication and scheduled follow-up control). Specific tools and delivery models that actively include and help patients with COPD to improve and subsequently maintain their symptom control and functional level are needed[14,19–23]. Only out-patient centered PR exists, and alternative rehabilitation and maintenance programs that overcome accessibility barriers do not exist[24]. The lack of public initiatives addressing this limited access to rehabilitation can contribute to inequality in health in already exposed patients with progressed COPD. Thus, there are good reasons to improve access and awareness of potential benefits from new delivery models of pulmonary rehabilitation and maintenance programs.

We use two evidence-based structured model of HPR and PTR with health-care supportive technology to meet these requirements and to test the clinical efficacy and model acceptance[14,19]. The aims of this study are to (1) compare the clinical benefits of the two rehabilitation models with 'usual care'; (2) investigate adherence to and effect from an exercise maintenance-program; (3) to qualitatively investigate the patients' experience with the intervention and maintenance program; (4) to investigate the program costs and explore the way in which the exercise programs are carried out and adapted. These aims are elaborated in the following section.

## *2.1 Objectives and hypotheses*

***2.1.1 Primary objective (RCT- study I)*** of the REPORT study is to evaluate the effects of structured home-based pulmonary rehabilitation (HPR) and supervised pulmonary tele-rehabilitation (PTR) on respiratory symptoms, quality of life, functioning and physical activity in patients with moderate to severe COPD who are unable or unwilling to access the conventional hospital- and community-based PR programs.

***2.1.2 Our hypotheses*** are that HPR and PTR will lead to respiratory symptom relief in the patients and that PTR and HPR will be equivalent to each other and superior to usual care, that is the a control group (CON - no intervention) among patients unable to access conventional rehabilitation programs.

***2.1.3 Aims for the overall research project***

1. To investigate equivalent effect of HPR and PTR and superiority to CON on patient-reported health parameters such as quality of life, anxiety-depression symptoms, sleep quality, fatigue, muscular-skeletal pain, physical activity and functioning level ***(RCT- study I) (table 2).***

2. Monitor and retrieve number of hospital admissions, length of stay and mortality from baseline throughout the follow-up period through the National Health Data Authorities ***(RCT- study I).***

3. To investigate adherence to and effect from an exercise maintenance-program (similar healthcare-technologies) on self-reported health parameters of quality of life, anxiety-depression symptoms, sleep quality, fatigue, muscular-skeletal pain, physical activity and functioning level (**Maintenance - study II).**

4. Qualitatively investigate the participant experience with the intervention and maintenance program and its impact on their everyday life involving social, structural, cultural and gender factors with the following aims.

# 3. Methodology – Study I and II

## *3.1 Design*

The REPORT study is designed as a multi-center three-arm randomized controlled trial, with a subsequent maintenance program.

## *3.2 Study population*

A total of 185 patients with moderate to very severe COPD who are unable to access the conventional hospital- and community-based PR programs when offered during routine consultations will be included from seven hospitals in the Capital Region, through general practitioners, healthcare centers and advertising through the Danish Lung Association to get a broad representative sample fulfilling our defined inclusion and exclusion criteria. During inclusion, baseline self-reported symptoms, functional capacity and physical activity is measured. Hereafter each participant is randomly assigned to one of the three arms in the study; HPR, PTR or CON. The HPR and PTR interventions begin within seven-to-ten days after randomization and patients participate in the 10-week assigned intervention and thereafter continue in a self-maintenance program for another 65-weeks (approx. 15-months). Self-reported respiratory symptoms constitute the primary outcome, assessed at the 10-week follow-up visit (primary endpoint). Follow-up assessment are done 10, 35 and 75 weeks (primary endpoint for the maintenance study) from baseline.

## *3.3 Recruitment and inclusion*

The practical aspects of the study and main research are carried out at the Respiratory Research Units, Department of Respiratory Medicine at Hvidovre, Bispebjerg and Herlev/Gentofte University Hospital and by their investigators, primarily postdoctoral fellow, PhD Henrik Hansen, Associate professor, MD Nina Godtfredsen, Associate professor, Associate professor, MD Torgny Wilcke, Associate professor, Senior researcher, Stig Mølsted, Professor, DSc., MD Charlotte Suppli Ulrik and with support from the departments' VIP, TAP and clinical staff.

***Please read "section 5 - recruitment of subjects and informed consent" for recruitment details.***

## *3.4 Criteria for participation*

### *3.4.1 Inclusion criteria*

- Indication for pulmonary rehabilitation according to national guidelines
- Unable to access and participate in the conventional out-patient hospital- or community-based PR when offered during routine consultation
- A post-bronchodilator ratio FEV1/FVC <70% (confirmed physician diagnosis of COPD)[7]
- A post-bronchodilator FEV1 <80% (degree of airway obstruction) corresponding to GOLD grade 2-4 (moderate to very severe)[7]
- GOLD group B, C, D corresponding to severe respiratory symptoms and/or frequent acute exacerbations[7]

- Able to stand up from a chair (height 44-46cm) and walk 10 meters independently (with or without a walking aid)
- Able to lift both arms to a horizontal level with a minimum of 1 kilogram's dumbbells in each hand

#### *3.4.2 Exclusion criteria*

- Participation in conventional PR in the past 24 months
- Cognitive impairment - unable to follow instructions
- Impaired hearing or vision – unable to see or hear instruction from a tablet
- Unable to understand and speak Danish
- Comorbidities where the exercise content is contraindicated
  (ie. treatment for diabetic foot ulcer, active cancer treatment, life expectancy <12-months)

### *3.5 Data collection, assessment, and follow-up (table 1)*

The primary daily project management is carried out by the research project-manager (Henrik Hansen). In addition, a project group (co-investigators), consisting of medical doctors and project nurses from the departments involved, will assist the project manager with the recruitment, sampling, and follow-up of patients. All medical decisions regarding patients will be taken by a medical doctor responsible for the treatment. Data is collected in RedCAP electronic Case Report Forms (eCRF), specific to each patient. Patients are continuously assessed against the study inclusion and exclusion criteria by the clinical staff (nurses, physicians).

The study data includes data from: socio-demographics, ethnicity, body composition, physical (function, activity, muscle strength, balance) outcomes as well as self-reported symptoms of anxiety, depression, fatigue, pain, respiratory symptoms, quality of life, sleep-quality and individual qualitative interviews concerning patient experience with intervention and maintenance program. Data from National Health Data Authorities on rehabilitation, rehospitalization, length of stay and mortality are included in the trajectory analysis.

#### *3.5.1 Measures obtained from the journal*

- Existing available data from lung function test.
- Home-oxygen therapy
- Prescribed medication
- Gender
- Age
- Marital status
- Smoking status
- Body mass index (otherwise measured)
- COPD classification GOLD 1-4 and A-D
- If heart failure classified by NYHA I-IV

#### *3.5.2 Measures obtained from the National Health Data Authorities at 75-weeks follow-up*

- Length of hospital stay.
- Number readmissions
- Mortality
- Any other received rehabilitation from inclusion (visit 0) to final assessment (visit 3) 75-weeks follow-up.

- Charlson comorbidity index

### *3.5.3 Self-reported outcomes (baseline and at all follow-up visits)*

- COPD Assessment Test (CAT) - (respiratory symptoms)
- Hospital Anxiety and Depression Scale (HADS) – (Emotional symptoms)
- EuroQol-5D-3L (EQ-5D-3L) – (Quality of life – generic)
- Brief Pain Inventory (BPI) – (Body pain- intensity/location)
- Multidimensional Fatigue Inventory (MFI-20) – (Perceived fatigue)
- Pittsburg Sleep Quality Index (PSQI) – (Sleep Quality)

Approximately ≈30 patients will additionally be invited to participate in individual qualitative interviews concerning their personal experiences with intervention and maintenance programs. Data is collected continuously during the intervention period (phase-1) and the maintenance period (phase-2).

### *3.5.4 Physical function outcomes (baseline and at all follow-up visits)*

- ActivePAL triaxial accelerometer (PAL) – (Physical activity)
- 6-minute walk test (6MWT) – (walking capacity)
- 30-second sit to stand (30-sec-STS) – (leg muscle strength)
- Short Physical Performance Battery and (SPPB) – (function and frailty)
- Hand-grip strength (Jamar dynamometer) – (muscle strength upper extremity)

***Please read appendix 1. for information on the test procedure from the physical functioning test (Danish)***

## *3.6 Sample size and statistics*

*(performed by biostatistician Thomas Kallemose, Clinical Research Centre, University Hospital Hvidovre)*

Three primary pairwise comparisons are made in the study, equivalence test between the two interventions and the superiority test for each intervention compared to the control group for the primary outcome of respiratory symptoms (COPD assessment test). Sample size estimation for the equivalence is based on a t-test with equivalence margin of -2.5 to 2.5 (clinically relevant difference), standard deviation (SD) of 4.5, power of 80% and significance level of 5%, and estimated 56 patients necessary in each group. For both, superiority tests a minimal clinical difference of 2.5, SD of 4.5, power of 80% and significance level of 5% were used in a two-sample t-test, with fixed sample of 56 patients in the intervention groups. This yields a necessary size of 36 patients in the control group. The sample size is increased by 25% in each group to account for expected withdrawal and loss of power from adjustment and thus a total of 185 (70/70/45) patients. Power estimations for all secondary outcomes are based on the decided inclusion of 185 patients, expected standard deviation and established minimal clinical important difference (MCID) **(Table 2).**

### *3.6.1 Statistical analyses plan*

Analyses of the outcomes will be performed by the trial statistician (Thomas Kallemose) who is blinded to the treatment allocations. Secondary analyses will be performed by Postdoctoral fellow, PhD Henrik Hansen.

Baseline data will be reported as averages and standard deviations (medians and interquartile ranges) or frequencies and proportions as appropriate. Data analyses of both primary and secondary endpoints will be performed using intention-to-treat analyses. Additionally, per-protocol analyses including patients who complete ≥70% of the PTR and HPR rehabilitation program will be performed. Reporting will follow the CONSORT (Consolidated Standards of Reporting Trials) Statement for non-pharmacologic trials. Differences between the groups' changes of primary and secondary outcomes (10-weeks from baseline; 35-weeks from baseline and 75-weeks from baseline) will be analyzed by mixed effect models. The models include adjustment for treatment groups, age, sex, BMI, $FEV_1$, Charlson Comorbidity Index and smoking status, and a random effect recruitment site. To account for possible regression to the mean effect, the baseline measure for the outcome will be included as a fixed effect variable in the models. Normal distribution of the model residuals is evaluated by inspection of Q-Q plots. All data are considered missing at random and because of this, the ignorability assumption for the likelihood estimator is used to account for missing data. The number of used datasets will be transparent. Survival are visualized by Kaplan-Meier curves. Group differences on number of patients being adherent, hospitalization and death are analyzed using chi-squared test. No interim analysis will be made. Statistical analyses are carried out using R 3.2.2 (R Foundation for Statistical Computing, Vienna, Austria). P-values of less than 0.05 are considered statistically significant.

## Outcomes

| **Table 1. Flowchart measurement** | | **Visit 0** Baseline | **Visit 1** End of intervention | **Visit 2** **Maintenance** | **Visit 3** **Maintenance** |
|---|---|---|---|---|---|
| | | Baseline | Week 10 | Week 35 | Week 75 |
| **Primary outcome** | | | | | |
| Respiratory symptoms | COPD Assessment Test (CAT) | X | X | X | X |
| **Secondary outcomes** | | | | | |
| ***Self-reported health questionnaires*** | | | | | |
| Anxiety and depression symptoms | Hospital Anxiety and Depression Scale (HADS) | X | X | X | X |
| Quality of life – generic | EuroQoL-5D-3L (EQ-5D-3L) | X | X | X | X |
| Body pain- intensity and location | Brief Pain Inventory (BPI) | X | X | X | X |
| Perceived mental and physical fatigue | Multidimensional Fatigue Inventory (MFI-20) | X | X | X | X |
| Sleep Quality | Pittsburg Sleep Quality Index (PSQI) | X | X | X | X |
| ***Physical activity and function*** | | | | | |
| Physical activity | ActivePAL triaxial accelerometer (PAL) | X | X | X | X |
| Walking capacity | 6-minute walk test (6MWT) | X | X | X | X |
| Leg muscle endurance | 30-second sit to stand (30-sec-STS) | X | X | X | X |
| Static and dynamic balance | Guralnic test (SPPB) and Timed-up-go (TUG) | X | X | X | X |
| Muscle strength upper extremity | Hand-grip strength (Jamar dynamometer) | X | X | X | X |
| Data registrations and expense indicators | Adherence to intervention/self-maintenance | | X | X | X |
| | Adverse events recorded | | X | X | X |
| | Number of hospital admission | past 12-mo | X | X | X |
| | Length of stay – hospital admission | past 12-mo | X | X | X |
| | Consultant visits | past 12-mo | X | X | X |
| | Mortality | | X | X | X |
| | Comorbidities | X | X | X | X |
| **Descriptive variable** | | | | | |
| Lung function test | FEV1/FVC ratio in % | X | | | X |
| | FEV1 (% predicted) | X | | | X |
| | DLCO (% predicted) | if available | | | if available |
| Anthropometries | Body Mass Index (BMI) | X | X | X | X |
| | Body weight (kg) | X | X | X | X |
| | Body height (cm) | X | X | X | X |
| | Fat Free Mass Index (FFMI) | if available | if available | if available | if available |
| Other information reported | Smoking status | X | X | X | X |
| | Medication prescribed | X | X | X | X |
| | Bone fractures | past 12-mo | X | X | X |
| **Qualitative data** | | | | | |
| Qualitative study | Interview<br>Survey<br>Observation | Continuously during the recruitment, intervention and maintenance period | | | |

**Table 2. Anticipated power on secondary outcomes – superiority to control**

| Variables | Instrument | Subscales | Cronbach's alpha | Hypothesized Difference/ SD (anticipated power) |
|---|---|---|---|---|
| **Anxiety and depression** | Hospital Anxiety and Depressions Scale (HADS) | HADS-A scale (0-21)<br>HADS-D scale (0-21) | HADS-A 0.83<br>HADS-D 0.82 | HADS-A 1.5/2.5 (0.85)<br>HADS-D 1.5/2.5 (0.85) |
| **Health-Related Quality of Life** | EuroQoL 5-Dimension Questionnaire (EQ-5D) | EQ5D-questionnaire (mobility, self-care, usual activities, pain/discomfort, and anxiety/depression)<br>Norm based utility score (-0.624-1.000)<br>EQ5D-VAS (0-100 millimeters) | Not relevant – only one question in each dimension | EQ5D-VAS 8/16 (0.81) |
| **Body pain- intensity and location** | Brief Pain Inventory (BPI) | BPI-magnitude domain<br>BPI-inference domain<br>BPI-intensity (0-100 millimeter) | BPI-M 0.91<br>BPI-I 0.94 | BPI-intensity 2/4 (0.71) |
| **Perceived mental and physical fatigue** | Multidimensional Fatigue Inventory (MFI-20) | General fatigue<br>Physical fatigue<br>Reduced activity<br>Reduced motivation<br>Mental fatigue<br>Fatigue total score | GF 0.83<br>PF 0.85<br>RA 0.79<br>RM 0.82<br>MF 0.91 | Exploratory |
| **Perceived sleep-quality** | Pittsburgh Sleep Quality Index (PSQI) | Sleep Quality – component 1<br>Sleep Latency – component 2<br>Sleep duration – component 3<br>Habitual sleep efficiency – component 4<br>Sleep Disturbance – component 5<br>Use of sleep medication – component 6<br>Daytime dysfunction – component 7<br>Global PSQI score | Sleep Quality 0.57<br>Sleep Latency 0.45<br>Sleep duration 0.36<br>Sleep efficiency 0.46<br>Sleep disturbance 0.36<br>Sleep medication 0.28<br>Daytime dysfunction 0.25<br>Global PSQI 0.69 | Exploratory |
| **Physical activity** | *active*PAL™ activity monitor (PAL Technologies Ltd., Glasgow, UK) | Steps per day<br>Minutes lying/sitting<br>Minutes standing/walking<br>Number of body transitions per day | Not relevant | Steps per day 1100/2262 (0.79) |
| **Walking capacity** | 6-minute walk test (6MWT) | Distance in meter | Not relevant | 26/44,5 (0.83) |
| **Muscle strength and endurance in legs** | 30 seconds sit-to-stand test | Total numbers of repetitions | Not relevant | 2.0/2.5 (0.98) |
| **Static dynamic balance** | Guralnic test,<br>Timed up and go,<br>4-meter gait speed | Time, seconds | Not relevant | Not established<br>1.4/2.4 (0.83)<br>0.11/0.13 (0.99) |
| **Hand-grip muscle strength** | Jamar hand-grip dynamometer | Kilo | Note relevant | 5.0/7.3 (0.93) |

#### *3.6.2 Randomization*

The randomization allocation to HPR, PTR, CON is performed using block randomization with a randomized block size of 6 and 12 in random order. The randomization list will be generated in REDCap by the trial statistician and is not accessible to investigators involved in the conduct of the study until the database has been unlocked.

After written informed consent and completion of study assessments, patients are randomized to either HPR, PTR or CON. The target number of patients randomized to each group is 70, 70 and 45 respectively. After identification of an eligible patient, the patient is assigned a randomization number as identifier throughout the study. The participants are informed about which group they are randomized to by email and phone call. The patients in the three groups begin their assigned intervention within seven to ten days after randomization.

### *3.7 Interventions (also read figures in appendices 1)*

#### *3.7.1 Pulmonary Tele-Rehabilitation (PTR)*[14]

The PTR intervention is supervised by skilled physiotherapists and respiratory nurses with at least 2-years of substantial experience with PR and delivered via a virtual platform at Bispebjerg and Hvidovre Hospital to a group of 4–6 patients who exercise at home and communicate via tablet with Center for IT and Medico (CIMT) approved communication software. Each session is 60 min; 35 min of exercise and 25 min of patient education, two times per week for the duration of 10 weeks. The specific exercises used are evidence-based as they have been used in several intervention studies on patients with severe or very severe COPD and involves larger muscle groups with 50/50% exercises for upper and lower extremities, respectively[4,14,19]. Volume, intensity and content specified for the exercise protocol is in accordance with both national and international exercise recommendations[4,5]. The exercise sessions include relative progression every fourth week, as the patients are expected to improve their mental and physical capacity during the intervention. Additionally, to the supervised program, patients are instructed to do daily physical activity of at least 30 minutes by modalities accessible to the patient, e.g. in- or outdoor walking activities in bouts of minimum 10 minutes.

Every second session of the patient education consists of 25 minutes of Mindfulness-based Cognitive Therapy (MCBT) exercises and education developed for COPD patients with the potential mediating effects of mindfulness, self-compassion and breathlessness catastrophizing[25]. The other education sessions consist of dialogue reflections around empowerment of the subjects: action-plan for symptom deterioration, medication-technic and correct use, better living with COPD, nutrition and importance of smoking cessation.

The physical exercises are available on the tablet as instructed exercise-film for self-initiated exercising. The mindfulness exercises are available from sound-files on the tablet for self-initiated mindfulness-guidance. Educational material is delivered in hard copies together with tablet and exercise equipment but also available as audio-book chapters on the tablet in layman language. The book is called Better Living with Chronic Obstructive Pulmonary Disease – a patient guide. Exercise equipment is a package of a step-box and pairs of dumbbells weight from 1 to 5 kilo (heavier dumbbells available on request).

### *3.7.2 Home-based Pulmonary Rehabilitation (HPR)*[19]

The HPR intervention is an individual self-initiated home-based PR program with motivational and professional counseling, supported by health technology e.g. telephone call and facetime with choice of preference. The first session is a home visit by an experienced respiratory physiotherapist/nurse. During the visit the physiotherapist/nurse works with the patient to establish exercise goals, write a formal exercise prescription of four to six exercises, provide a mindfulness program and education in use of a home diary and assess inhaler technique. The physiotherapist also supervises the first exercise and mindfulness session. The patient is informed that performing the self-initiated exercise and mindfulness program is likely to improve their symptoms, fitness and physical functioning.
The overall goal is for the patient to achieve at least 30 minutes of self-initiated muscle-endurance based exercise three days a week. The prescribed physical exercises are available on the tablet as an instructed exercise-film. Secondly the patient is instructed to complete a mindfulness program of choice for 15 minutes at least once a week. Seven mindfulness exercises are available from sound-files on the tablet for self-initiated mindfulness-sessions.

Additionally, daily physical activity of at least 30 minutes by modalities accessible to the patient, e.g. in- or outdoor walking activities in bouts of minimum 10 minutes. During the initial visit the physiotherapist/nurse accompanies the participant on their initial walk (weather permitting for those who had chosen to walk outside the home). This allows planning of an appropriate route around the local neighborhood consistent with the patient's abilities (e.g. ability to walk stairs/hills).

In a home diary the patient is instructed to record when, where and how often she/he plans to perform exercise and mindfulness; what might get in the way of the plans this week; and what can be done to overcome any perceived barriers. The diary includes a telephone number so the patient can contact the physiotherapist/nurse between sessions if required to answer questions related to the program. During the initial home visit the physiotherapist/nurse also assesses safety of the home environment in relation to the exercise program.

The home visit is followed by once weekly structured web/phone call from the physiotherapist/nurse for 10-weeks using a motivational interviewing approach. During the telephone calls, the patient discusses the exercise, mindfulness program and exercise goals, and have the option to discuss other health issues in relation to COPD. A typical telephone call will begin with a brief talk of what has happened during the exercise program over the past week. Consistent with the principles of motivational interviewing, the physiotherapist/nurse might then ask the patient why she/he might want to increase the exercise program (desire); how she/he might do this if she/he decides to (ability); what would be the most important benefits in doing more exercise (reasons); and how important it is for the patient to do more exercise at this time (need). The physiotherapist will then encourage the participant to move towards commitment and action, with specific goal set for the following week. During each web/phone call the participants document their goals in their diaries in the same manner as from the initial home visit session. The discussion will then move on to other issues in relation to COPD. The patient is provided with a menu of topics relevant to COPD and self-care and encouraged to select a topic of relevance for discussion, providing opportunity for self-management. Education materials are delivered in hard copies together with the tablet and exercise equipment but also available as audio-book chapter on tablet in layman language. The book is called Better Living with Chronic Obstructive Pulmonary Disease – a patient guide. Exercise equipment is a package of a step-box and pairs of dumbbells weight from 1 to 5 kilo (heavier dumbbells available on request).

#### *3.7.3 Control*

Patients randomized to the control group will receive usual care; medication, scheduled follow-up visit and possible phone contact with GP or the outpatient respiratory department. Except for assessment visits 10-, 35-, and 75-weeks from baseline no intervention is offered.
If a patient changes his/her mind and wishes to participate in a conventional hospital- or community-based PR program, it will be granted as this is a highly recommended treatment (e.g. rehabilitation after hospital admitted exacerbation).

#### *3.7.4 Maintenance*[26]

During the 15-month maintenance program, patients are asked to continue at home with a similar program to that completed. The weekly goal is for the patient to achieve at least 30 minutes of self-initiated muscle-endurance based exercises twice weekly, complete a mindfulness program of choice for 15 minutes at least once a week and, in addition they are once weekly offered healthcare professional (physiotherapist/nurse) supervised exercise (groups of 4-6 persons), mindfulness and dialogue/consultation for 60 minutes throughout the 15-month maintenance period.
The prescribed physical exercises are available on the tablet as an instructed exercise-film. Seven mindfulness exercises are available from sound-files on the tablet for self-initiated mindfulness-guidance.

The overall goal for the maintenance program is that the patient achieves sustainable exercise habits and maintain or even improve gains from the intervention.

#### *3.7.5 Use of hardware and software technology for intervention delivery*

The Capital Region of Copenhagen will provide android tablets with the pre-developed tools/e-tools, online exercise programs, internet installed and provide delivery and full service including the exercise equipment.

The system is approved by Center for IT and Medico-Technology (CIMT) and the data protection agency. The screen is a Samsung tablet touch screen, with a single interface that can be operated by all patients. The screen for healthcare professionals consists of a professional video conferencing system that makes it possible to see multiple patients during a training session and train the group synchronously in real time. The product supports the possibility of group training via video conference and is already used for other telemedical consultations.

The research staff has in-dept knowledge and experience with the technology. From our previous telerehabilitation study, we documented that major technical issues leading to cancellation and rescheduling of group sessions affected 2 of 360 group sessions[14]. Minor temporary technical issues (that is sound artefacts, screen freezes) not leading to cancellation or delay were present in 14% of the total group session (49 of 360). Individual patient cancellation caused by technical problems was 12 of 1902 individual connections.[14]

## 4. Risk, harms, and side effects

As mentioned above, pulmonary rehabilitation is considered cornerstone treatment to patient with severe COPD[4–6]. Even though COPD leads to profound pathophysiological changes, exercise training has minimal risks. The risks associated with participation in the REPORT study are considered minimal, as both interventions are developed based on current guideline recommendations on physical exertion, exercise and activity for patients with COPD[4–6].

We are not aware of any serious circumstances (death, hospitalization, serious injury related to the functional tests) from clinical studies or clinical practice in related patient groups that have undergone similar functional test which has led to termination of the trial as a whole or any specific functional test. Subjects should anticipate the possibility of muscle soreness that typically lasts one to two days after assessments. Neither the examination nor the physical tests are more intrusive to regular out-patient examination after respiratory exacerbations. A few safety precautions described below are applied for the participants in the study.

### *4.1 Assessment of safety*

Information about adverse events and serious adverse events related to the interventions, whether reported by the participant, discovered by the researchers by reviewing medical records, detected through examinations during assessment-visits, through tests or other means, that occur from the time the informed consent form is signed by the patient and until assessment-visit 3 will be recorded and reported on an adverse event page in the e-CRF. Evaluation of adverse events and serious adverse events, including severity, causality, outcome and seriousness assessments, will be performed by a senior physician (Charlotte Ulrik, Torgny Wilcke and Nina Godtfredsen) and reviewed by the international advisory board.

## 5. Recruitment of subjects, informed consent

### *5.1 Recruitment*

We will recruit through seven departments of respiratory medicine (Amager, Hvidovre, Bispebjerg, Herlev, Gentofte, Frederikssund and Hillerød University Hospitals) in the Capital Region of Copenhagen, through general practitioners and community healthcare centers to get a broad representative sample from the target population. Patients are continuously assessed against the study inclusion and exclusion criteria by the clinical staff (nurses or physicians).

The first invitation to this project takes place when patients are unable to access the conventional hospital- and community-based PR programs when offered during routine consultations and when inclusion and none of the exclusion criteria is confirmed by the clinical staff (nurses or physicians). The clinical staff receives patient consent to contact investigator. Subsequent the investigator will contact the patient 3-7 days after receiving the consent to contact from the clinical staff. Recruitment letter and participant information are post mailed to patient who consent to be contacted by the investigator. Recruitment letter and information material are also possible for the clinical staff to provide on beforehand if requested by the patient.

If a patient is suitable, the person will be invited to participate in the project. Participation in the trial is voluntary. Informed consent is obtained from the participants by executive order no. 1149, 30th September 2013 with information and consent for participation in health science research projects as well as on notification of health science research projects.

The participant information is provided both orally and in writing, and the patient is informed that they are entitled to 24 hours of consideration before consent is given for participation in the trial. Participants who wish to participate after consideration may give written consent. The oral information is provided by the investigator or project staff who has the required skills and who is directly related to the project. The right of occupant is ensured by the patient being able to bring a bystander. If no bystanders participate when oral information is given, they are ensured, afterwards, whenever possible. It is ensured that the conversation is undisturbed by using undisturbed consultation room/phone-call/ patients home, decided by patient convenience and choice of preference. The research subject will be given the document " Forsøgspersonens

rettigheder i et sundhedsvidenskabeligt forskningsprojekt ", which contains information about confidentiality, access to documents and access to complaints. The subjects are protected under the Data Protection Regulation/Databeskyttelsesforordningen and the Data Protection Act/ Databeskyttelsesloven. The trial has been reported to the Regional Science Ethics Committee, and approved by the Danish Data Protection agency (P-2022-245).

It must be ensured at all times that subjects have consented to participate in the trial.

### *5.2 Enrollment*

Approximately 1,100 patients are offered conventional hospital- or community based pulmonary rehabilitation during routine consultations annually in the Capital Region of Copenhagen. From a previous study, we know that a least 700 patients decline participation. From our ongoing cross-sectional preparation study (clinicalTrials.gov NCT04249388) we have collected data suggesting 3 out of 5 are willing to be included in the REPORT study, and therefore it should be possible to recruit and include 185 patients within a pre-set timeframe of 18 to 24-months.

We strive to limit the rate of patients who discontinue and limit loss-to-follow-up by being flexible in relation to scheduling of intervention sessions and visits/assessments will be offered at patient residences or local hospitals by choice of preference. Further, we aim to constantly adjust the individual goal setting and action plan to fit the patient’s physical abilities. If a patient discontinues, she/he is encouraged to keep following the instructions given and to attend the remaining follow-up assessment to allow us to collect follow-up data.

## 6. Availability of information and right to data:

The consent gives the investigator and any control authority direct access to obtain information in the patient's record, including electronic records, in order to see information about the subject's health conditions which are necessary as part of conducting the research project and for control purposes, including safety, quality control and monitoring which are required.

The investigator group that has designed and conducted this study has the right to data and the right (and duty) to publish based on data. All sites that recruit patients are entitled to at least one authorship on the primary publication. It is the opinion of the investigator group that knowledge sharing creates more and better scientific results. Requests for knowledge sharing from other groups or request to do sub studies will be assessed individually and only by approval from the Danish Data Protection Agency.

PhD students and master students doing their thesis in the setting of the study will be granted access to use the data by the Danish Data protection Agency and the core scientific research group.

### *6.2 data storage*

An electronic case report form (Research Electronic Data Capture, REDCap) is provided and all data related to the study are recorded providing the basis for a central database. In this central database, data are stored in coded form according to the rules of the Danish Data Protection Agency (Datatilsynet). The electronic case report form (e-CRF) is to be completed by blinded assessors at the time of the participant’s assessments time-points, so that it always reflects the latest observations for the patient. Data will be stored for 10 years, after which they will be transferred to The Danish National Archives (“Rigsarkivet”). Source data are registered directly in the e-CRF.

### *6.2 Approvals*

The study has been approved by the Danish Data Protection agency (P-2022-245) and the Scientific Ethics Committee of the Capital Region (H-22015777).

## 7. Ethical report

The study is conducted according to the Declaration of Helsinki and is carried out in accordance with the rules of the Act personal data and the Health Act. The study has been approved by the Danish Data Protection agency (P-2022-245). The study will be registered with the clinical database, ClinicalTrials.gov before initiation and the first inclusion. Recruitment and inclusion will take place as previously described *(section 5)*. Participation requires one signed consent declaration. Patients can withdraw their participation consent at any time without affecting their right to present or future treatment. The patient is also entitled to bring a bystander to the information interview and is entitled to report time before any declaration of consent is signed.

The important objective of the study is to investigate the effects of structured home-based pulmonary rehabilitation (HPR) and supervised pulmonary tele-rehabilitation (PTR) on respiratory symptoms, quality of life, functioning and physical activity in patients with moderate to severe COPD who are unable or unwilling to access the conventional hospital- and community-based PR programs. That includes investigating if HPR and PTR will be equivalent to each other and superior to usual care, that is the control group (CON – medical visits) among patients unable to access conventional rehabilitation programs. Potential disadvantages and side effects are described in separate section 4. Here it appears, inter alia, that the likelihood of serious adverse reactions to the functional test and intervention is very rare.

By participating in the study, the individual patient is given the opportunity to engage in a pulmonary rehabilitation intervention free of charge and expanded access to a rehabilitation program usually restricted to centralized hospitals and community healthcare centers. In addition, the patient is provided with relevant health information related to themselves and their disease.

The method and statistical analyzes have been carefully considered to be able to confirm or reject the hypotheses for this study. true representative averages and to reassess changes at 52-weeks follow-up.

In the light of the above considerations, we believe that the study is ethically sound and can be completed without exposing the test participant to unjustifiable risks.

## 8. Time-schedule and budget

The REPORT study is scheduled to begin recruitment of patients in August 2022 and is expected to continue recruitment until March 2024 and, therefore planned to finish the final 15-month maintenance assessments no later than January 2026. The practical aspects of REPORT include recruitment, inclusion, intervention and assessments of primary and secondary endpoints, as illustrated in the green section of the project timeline, and should be finished within one and a half year (short-term rehabilitation) and after three and a half years (long-term maintenance; 75-week follow-up visit, January 2026) from 'first patient first visit' (figure 1).

The research study is investigator initiated by Henrik Hansen and Nina Godtfredsen from the Respiratory Research Units, Department of Respiratory Medicine, Hvidovre. The study is funded by a total of 5.482 mio. kr (Trygfonden: 2.957mio kr.; Capital Region: 0.600mio kr.; Danske Fysioterapeuter: 0.450 mio kr.; Jascha Fonden: 0.400 mio kr.; Lundbeck fonden: 0.300 mio kr.; Telemedical Center Capital Region: 0.250 mio kr.; Lungeforeningen: 0.225 mio kr.; VIP salaries: 0.300 mio kr.) used for salary to one postdoctoral fellow (Henrik Hansen), one PhD student (to be assigned), one research nurse (Lisbeth Østergaard), TAP personal

(clinical staff), blinded assessors (clinical staff), equipment, tablets, publication and conference fees etc. No core clinical group member has relations to private companies or funding sources with conflict of interest in relation to the research study.

## 9. Remuneration

No remuneration is paid to the participants.

## 10. Dissemination of results

Positive, negative, and inconclusive findings will be submitted for publication in highly impact international peer-reviewed scientific journals, e.g. Thorax, European Respiratory Journal, and Respiratory Medicine. The results will be presented and discussed at international.

The findings from the study will also be disseminated in information material to the public (for example in flyers exposed in the general practitioners' waiting rooms).

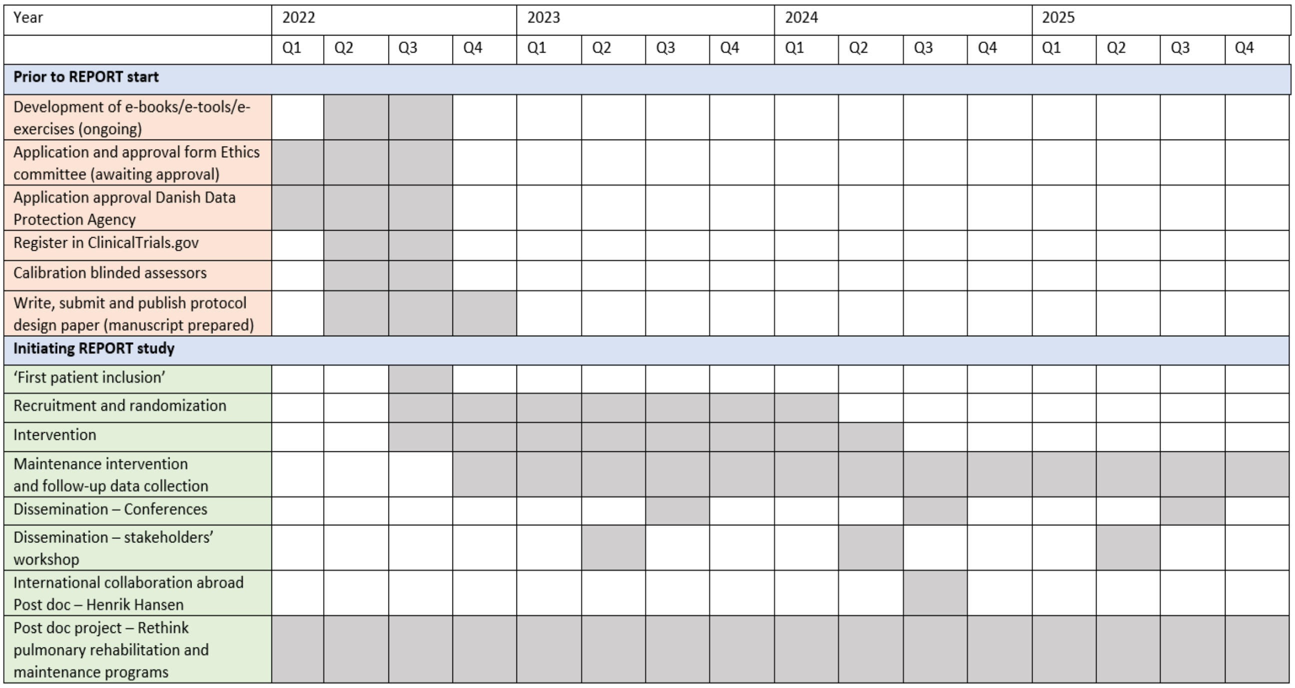


**Figure 1. Projected timeline**

## 11. Information on compensation or reimbursement schemes:

Patients who participate in the study and who believe they have suffered injury can seek compensation through the patient compensation (http://patienterstatningen.dk/). Applicable by Danish law.

## 12. Research Organization and Investigators

The REPORT study is headed by postdoctoral fellow, PhD, PT. Henrik Hansen, Associate research professor, PhD, MD Nina Skavlan Godtfredsen and Professor, DMsc., MD, Charlotte Suppli Ulrik and carried out in

collaboration between the pulmonary departments at North Zealand, Gentofte-Herlev, Bispebjerg and Amager-Hvidovre University Hospitals.

The practical aspects of the study are managed at the Respiratory Research Unit, Department of Respiratory Medicine at Hvidovre Hospital and Department of Respiratory Medicine at Bispebjerg Hospital. Postdoctoral fellow Henrik Hansen will have the overall responsibility for the daily practical work. He has 16 years of experience with pulmonary rehabilitation PR including alternative delivery models and possesses the required experienced with similar multi-center project structure from a successful project completion during his PhD enrollment. He will lead a group consisting of a research nurse (to be assigned), a PhD student (to be assigned) and research assistants (MD-students, Public health student), blinded project assessors and intervention staff (part-time employed from clinic).

The senior researchers, Associate Research Professor Nina Skavlan Godtfredsen, Associate Professor Stig Mølsted, Associate Professor Torgny Wilcke and Professor Charlotte Suppli Ulrik will ensure and be responsible for the overall scientific quality and progress of the project. The established national and international advisory board will oversee the quality and validity of this research project.

## *12.1 Investigators (Core Clinical study group)*


- Research responsible and project leader, Postdoctoral fellow, PhD, PT. Henrik Hansen

  Respiratory Research Units, Department of Respiratory Medicine, Hvidovre

  Phone Tlf.: 2894 6780
  Mail: henrik.hansen.09@regionh.dk

- Associate professor, MD Nina Godtfredsen,

  Respiratory Research Units, Department of Respiratory Medicine, Hvidovre
  Tlf.: 3862 6310
  Mail: Nina.Skavlan.Godtfredsen@regionh.dk

- Associate professor, MD Torgny Wilcke

  Respiratory Research Units, Department of Respiratory Medicine, Herlev and Gentofte
  Mail: t.wilcke@dadlnet.dk

- Associate professor, Senior researcher, Stig Mølsted,

  Department for clinical research and department of Physiotherapy
  Mail: Stig.Moelsted@regionh.dk

- Professor, DSc., MD Charlotte Suppli Ulrik

  Respiratory Research Units, Department of Respiratory Medicine, Hvidovre
  Mail: csulrik@dadlnet.dk

## *12.2 National Steering committee*

Henrik Hansen, research responsible trial manager, postdoctoral fellow, PhD, PT, Respiratory Research Unit, Dept. of respiratory Medicine, Copenhagen University Hospital Hvidovre, Denmark

Nina Skavlan Godtfredsen, supervisor, MD, PhD, Associate Professor, Rehabilitation responsible, Respiratory Research Unit, Dept. of respiratory Medicine, Copenhagen University Hospital Hvidovre, Denmark and Institute of Clinical Medicine, University of copenhagen Faculty of Health and Medical Sciences, Copenhagen, Denmark

Stig Mølsted, Senior Researcher, Associate professor, PhD, PT, Department of Clinical Research, Copenhagen University Hospital North Zealand, Hillerød, Denmark

Nina Beyer, Emerita Associated professor, PhD, Institute for clinical Medicine, University of Copenhagen Faculty of health and Medical sciences, Copenhagen, Denmark

Theresa Bieler, Postdoctoral fellow, PhD, PT, Department of Physical and Occupational Therapy, Copenhagen University Hospital Bispebjerg, Copenhagen, Denmark

Gerd Martinez, PT, Department of Respiratory Medicine, Copenhagen University Hospital Hvidovre, Copenhagen, Denmark

Torgny Wilcke, MD, PhD, Associate Professor, Rehabilitation responsible, Dept. of Respiratory Medicine, Copenhagen University Hospital Gentofte

Marie Lavesen, RN, Emergency Department, Copenhagen University Hospital North Zealand, Hillerød, Denmark

Anja Berill, RN, Dept. of respiratory Medicine, Copenhagen University Hospital North Zealand, Frederikssund, Denmark

Lisbeth Østergaard, RN, Dept. of Respiratory Medicine, Copenhagen University Hospital Bispebjerg, Denmark

Helle Frost Andreassen, MD, Head of department, MPG, PhD, Dept. of Respiratory Medicine, Copenhagen University Hospital Bispebjerg, Denmark

Hanne Ehlers, RN, Head of nursing, Dept. of Respiratory Medicine, Copenhagen University Hospital Bispebjerg, Denmark

Judith Lørup Rindum, Head of telemedical knowledge Center, Capital Region of Copenhagen

Charlotte Suppli Ulrik, Clinical Chair Professor, MD, DMsc, Head of Respiratory Research Unit Hvidovre, Dept. of Respiratory Medicine, Copenhagen University Hospital Hvidovre, Denmark and Institute of Clinical Medicine, Faculty of Health and Medical Sciences, Copenhagen, Denmark

## *12.3 International Advisory Board*

The international advisory board will oversee the data collection, intervention delivery, progression, safety and finally the interpretation of the results.

The international board will attend 2 meetings annually via videoconference-meetings or scheduled physical meetings at the annually international conferences at the American Thoracic Society (ATS) in May and European Respiratory Society (ERS) in September. The surveillance meetings days in Copenhagen will consist

of three times two days in the period from second half of 2022 to first half of 2025. All travel expenses for surveillance will be covered within the research budget, while the advisory board members are unremunerated.

The international board members are internationally recognized elite researchers within pulmonary rehabilitation and medicine and consist of:

- Therese Lapperre, Professor, PhD, MD, Head of Dept Pulmonology, University Hospital Antwerp, Antwerp, Belgium and Laboratory of Experimental Medicine and Paediatrics, University of Antwerp, Antwerp, Belgium.

  Therese Lapperre is a specialist MD in pulmonary medicine and PhD, involved in several international RCT studies. She worked as MD and Associate professor in Denmark until the beginning of 2020, before continuing her career as research professor and head of the pulmonary department at university hospital in Antwerp, Belgium. Therese Lapperre understands the Danish healthcare system and procedures and will contribute with her medical skills and perspectives for our study execution.
- Martijn Spruit, Professor, PhD, PT, Board director, FERS
  Department of Respiratory Medicine, School of Nutrition and Translational Research in Metabolism, Faculty of Health, Medicine and Life Science, Maastricht University, the Netherland
  and Dept of Research and Education, CIRO+, Center of Expertise for Chronic Organ Failure, Horn, the Netherlands.

- Anne Holland, Professor, PhD, PT
  Head of Respiratory Research at Alfred Health hospital and Monash University, Melbourne, Australia

Martijn Spruit and Anne Holland have a background as physiotherapists. They are both highly acknowledged for their research contribution within the area of pulmonary rehabilitation, muscle skeletal functioning, oxygen and respiratory therapy. Together they have coauthored the most important research articles within the area of pulmonary rehabilitation for the past 10 years have been involved in more than 40 RCT studies.

## References


1. Sundhedsdatastyrelsen. Kronisk obstruktiv lungesygdom (KOL) - Sundhedsdatastyrelsen. Sundhedsdatastyrelsen. Published 2020. Accessed August 14, 2020. https://sundhedsdatastyrelsen.dk/da/tal-og-analyser/analyser-og-rapporter/sygdomme/kronisk_obstruktiv_lungesygdom

2. Hansen JG, Pedersen L, Overvad K, Omland Ø, Jensen HK, Sørensen HT. The prevalence of chronic obstructive pulmonary disease among danes aged 45-84 years: Population-based study. *COPD J Chronic Obstr Pulm Dis*. 2008;5(6):347-352. doi:10.1080/15412550802522635

3. Davidsen;, Anne Illemann Christensen; Michael, Ola Ekholm, Pia Vivian Pedersen KJ. *Danskernes Sundhed-Den Nationale Sundhedsprofil*.; 2013. Accessed August 14, 2020. www.sst.dk

4. Spruit MA, Singh SJ, Garvey C, et al. An official American thoracic society/European respiratory society statement: Key concepts and advances in pulmonary rehabilitation. *Am J Respir Crit Care Med*. 2013;188(8). doi:10.1164/rccm.201309-1634ST

5. Danish National board of Health. *National Klinisk Retningslinje for Rehabilitering Af Patienter Med KOL*.; 2018. Accessed October 2, 2018. https://www.sst.dk/da/udgivelser/2018/~/media/AD2FF426014943D983E0D7B937B356B9.ashx

6. McCarthy B, Casey D, Devane D, Murphy K, Murphy E, Lacasse Y. Pulmonary rehabilitation for chronic obstructive pulmonary disease. *Cochrane Database Syst Rev*. 2015;2015(2):1-209. doi:10.1002/14651858.CD003793.pub3.www.cochranelibrary.com

7. Agusti A, Hurd S, Jones P, Fabbri LM, Martinez F VC et al. *Global Strategy for the Diagnosis, Management, and Prevention of Chronic Obstructive Pulmonary Disease*.; 2020. Accessed August 28, 2020. www.goldcopd.org

8. Spruit MA, Pitta F, Garvey C, et al. Differences in content and organisational aspects of pulmonary rehabilitation programmes. *Eur Respir J*. 2014;43:1326-1337. doi:10.1183/09031936.00145613

9. Rochester CL, Vogiatzis I, Holland AE, et al. An Official American Thoracic Society/European Respiratory Society Policy Statement: Enhancing Implementation, Use, and Delivery of Pulmonary Rehabilitation. *Am J Respir Crit Care Med*. 2015;192(11):1373-1386. doi:10.1164/rccm.201510-1966ST

10. Keating A, Lee A, Holland AE. What prevents people with chronic obstructive pulmonary disease from attending pulmonary rehabilitation? A systematic review. *Chron Respir Dis*. 2011;8(2):89-99. doi:10.1177/1479972310393756

11. Fischer MJ, Scharloo M, Abbink JJ, et al. Drop-out and attendance in pulmonary rehabilitation : The role of clinical and psychosocial variables. *Respir Med*. 2009;103(10):1564-1571. doi:10.1016/j.rmed.2008.11.020

12. Bjoernshave B, Korsgaard J, Jensen C, Vinther Nielsen C. Participation in pulmonary rehabilitation in routine clinical practice. *Clin Respir J*. 2011;5(4):235-244. doi:10.1111/j.1752-699X.2011.00237.x

13. Bjoernshave B, Korsgaard J, Nielsen CV. Does pulmonary rehabilitation work in clinical practice? A review on selection and dropout in randomized controlled trials on pulmonary rehabilitation. *Clin Epidemiol*. 2010;2:73-83. Accessed February 12, 2017. http://www.ncbi.nlm.nih.gov/pubmed/20865106

14. Hansen H, Bieler T, Beyer N, et al. Supervised pulmonary tele-rehabilitation versus pulmonary rehabilitation in severe COPD: A randomised multicentre trial. *Thorax*. 2020;75(5):413-421. doi:10.1136/thoraxjnl-2019-214246

15. Johnston K, Young M, Grimmer K, Antic R, Frith P. Frequency of referral to and attendance at a pulmonary rehabilitation program amongst patients admitted to a tertiary hospital with chronic obstructive pulmonary disease. *Respirology*. 2013;18(7):n/a-n/a. doi:10.1111/resp.12128

16. Godtfredsen N, Frølich A, Bieler T, et al. 12-months follow-up of pulmonary tele-rehabilitation versus standard pulmonary rehabilitation: A multicentre randomised clinical trial in patients with severe COPD. *Respir Med*. 2020;172:106129. doi:10.1016/j.rmed.2020.106129

17. Cox NS, Oliveira CC, Lahham A, Holland AE. Pulmonary rehabilitation referral and participation are commonly influenced by environment, knowledge, and beliefs about consequences: a systematic review using the Theoretical Domains Framework. *J Physiother*. 2017;63(2):84-93. doi:10.1016/j.jphys.2017.02.002

18. Casaburi R. A Brief History of Pulmonary Rehabilitation Introduction History of Pulmonary Rehabilitation Alvan L Barach MD Thomas L Petty MD The Dark Ages Resurgence of Pulmonary Rehabilitation Summary. *Care*. 2008;53(9):1185-1189. Accessed December 18, 2018. http://www.rcjournal.com/contents/09.08/09.08.1185.pdf

19. Holland AE, Mahal A, Hill CJ, et al. Home-based rehabilitation for COPD using minimal resources: A randomised, controlled equivalence trial. *Thorax*. 2017;72(1):57-65. doi:10.1136/thoraxjnl-2016-208514

20. Cox NS, Dal Corso S, Hansen H, McDonald CF, Hill CJ, Zanaboni P, Alison JA, O'Halloran P, Macdonald H HA. Telerehabilitation for chronic respiratory disease. *Cochrane Database Syst Rev*. 2021;(1). doi:10.1002/14651858.CD013040

21. Rochester CL, Vogiatzis I, Holland AE, et al. An official American Thoracic Society/European Respiratory Society policy statement: Enhancing implementation, use, and delivery of pulmonary rehabilitation. *Am J Respir Crit Care Med*. 2015;192(11):1373-1386. doi:10.1164/rccm.201510-1966ST

22. Imamura S, Inagaki T, Terada J, Nagashima K, Katsura H, Tatsumi K. Long-term efficacy of pulmonary rehabilitation with home-based or low frequent maintenance programs in patients with chronic obstructive pulmonary disease : a meta-analysis Long-term efficacy of pulmonary rehabilitation with home-based or low frequent mai. *Ann Palliat Med*. 2020;1(August). doi:10.21037/apm-19-581

23. Malaguti C, Dal Corso S, Janjua S, Holland AE. Supervised maintenance programmes following pulmonary rehabilitation compared to usual care for chronic obstructive pulmonary disease. *Cochrane Database Syst Rev*. 2021;2021(8). doi:10.1002/14651858.CD013569.PUB2

24. Capital Region D. *Forløbsprogram for KOL Hospitaler, Almen Praksis Og Kommunerne i Region Hovedstaden*.; 2015. Accessed February 12, 2017. https://www.regionh.dk/Sundhedsaftale/bilag-og-download/Documents/RH_Program_KOL_rev_2015.pdf

25. Farver-Vestergaard I, O'Toole MS, O'Connor M, et al. Mindfulness-based cognitive therapy in COPD: A cluster randomised controlled trial. *Eur Respir J*. 2018;51(2):1702082. doi:10.1183/13993003.02082-2017

26. Vasilopoulou M, Papaioannou AI, Kaltsakas G, et al. Home-based maintenance tele-rehabilitation reduces the risk for acute exacerbations of COPD, hospitalisations and emergency department visits. *Eur Respir J*. 2017;49(5). doi:10.1183/13993003.02129-2016

27. Cane J, O'Connor D, Michie S. Validation of the theoretical domains framework for use in behaviour change and implementation research. *Implement Sci*. 2012;7(1):37. doi:10.1186/1748-5908-7-37

28. O'Brien B, Harris I, Beckman T, Reed D, Cook D. Standards for reporting qualitative research: a synthesis of recommendations. *Acad Med*. 2014;89(9):1245-1251. doi:10.1097/ACM.0000000000000388

29. von Elm E, Altman D, Egger M, Pocock S, Gøtzsche P, Vandenbroucke J. The Strengthening the Reporting of Observational Studies in Epidemiology (STROBE) statement: guidelines for reporting observational studies. *Lancet (London, England)*. 2007;370(9596):1453-1457. doi:10.1016/S0140-6736(07)61602-X

30. Kottner J, Audige L, Brorson S, et al. Guidelines for Reporting Reliability and Agreement Studies (GRRAS) were proposed. *Int J Nurs Stud*. 2011;48(6):661-671. doi:10.1016/j.ijnurstu.2011.01.016

31. Hansen H, Beyer N, Frølich A, Godtfredsen N, Bieler T. Intra- and inter-rater reproducibility of the 6-minute walk test and the 30-second sit-to-stand test in patients with severe and very severe COPD. *Int J Chron Obstruct Pulmon Dis*. 2018;Volume 13:3447-3457. doi:10.2147/COPD.S174248

32. Hansen H, Beyer N, Frølich A, Godtfredsen N, Bieler T. Inter-Day Test–Retest Reproducibility of the CAT, CCQ, HADS and EQ-5D-3L in Patients with Severe and Very Severe COPD. *Patient Relat Outcome Meas*. 2021;Volume 12:117-128. doi:10.2147/PROM.S306352

33. Mokkink LB, Boers M, van der Vleuten CPM, et al. COSMIN Risk of Bias checklist for systematic reviews of Patient-Reported Outcome Measures. 2018;27:1171-1179. doi:10.1007/s11136-017-1765-4

34. Weir JP. Quantifying Test-Retest Reliability Using the Intraclass Correlation Coefficient and the SEM. *J*

*Strength Cond Res*. 2005;19(1):231-240. Accessed July 24, 2018. http://citeseerx.ist.psu.edu/viewdoc/download?doi=10.1.1.457.2590&rep=rep1&type=pdf

35. De Vet HCW, Terwee CB, Knol DL, Bouter LM. When to use agreement versus reliability measures. *J Clin Epidemiol*. 2006;59(10):1033-1039. doi:10.1016/j.jclinepi.2005.10.015

36. Glick HA, Doshi JA, Sonnad SS, Polsky D. Economic Evaluation in Clinical Trials. *Econ Eval Clin Trials*. Published online October 28, 2014. doi:10.1093/MED/9780199685028.001.0001

37. Drummond, MF, Sculpher, MJ, Claxton, K, Stoddart, GL & Torrance G. *Methods for the Economic Evaluation of Health Care Programmes - Research Database, The University of York*. 4th ed. Oxford University Press; 2015. Accessed August 24, 2021. https://pure.york.ac.uk/portal/en/publications/methods-for-the-economic-evaluation-of-health-care-programmes(8f69bcee-cdac-44fa-871c-f821470df60a)/export.html

38. Manca A, Hawkins N, Sculpher M. Estimating mean QALYs in trial-based cost-effectiveness analysis: the importance of controlling for baseline utility. *Health Econ*. 2005;14(5):487-496. doi:10.1002/HEC.944

39. Husereau D, Drummond M, Petrou S, et al. Consolidated Health Economic Evaluation Reporting Standards (CHEERS) Statement. Published online 2013. doi:10.1016/j.jval.2013.02.010

## Appendices 1

| Intervention | Content - in brief |
|---|---|
| **Pulmonary Tele-Rehabilitation (PTR)** | • The PTR intervention is supervised by skilled physiotherapists and respiratory nurses.<br>• Delivered via a webcam CIMT approved communication software from Bispebjerg Hospital<br>• In groups of 4–6 patients; exercise at home and communicate via tablet for 10 weeks<br>• Each session is 60 min; 35 min of exercise and 25 min of patient education/ mindfulness<br>• Patients are instructed to do daily physical activity of at least thirty minutes of physical activity e.g. in- or outdoor walking activities of in bouts of minimum 10 minutes.<br>• Every second session consists of 25minutes of Mindfulness-based Cognitive Therapy (MCBT) exercises and education developed for COPD patients with the potential mediating effects of mindfulness, self-compassion, breathlessness, catastrophizing. The education session consists of dialogue reflections around empowerment of the patients: action-plan with symptom increase, medication-technic and correct use, better living with COPD, nutrition, importance of smoking cessation.<br>• The physical exercises are available on the tablet as an instructed exercise-(film)video for self-initiated exercising. The mindfulness exercises are available from sound-files on the tablet for self-initiated mindfulness-guidance.<br>• Education materials are delivered in paperback together with tablet and exercise equipment but also available as audio-book chapter on tablet in layman language.<br>• Exercise equipment is a package containing a step-box and pairs of dumbbells weight from 1 to 5 kilo (heavier dumbbells available on request). |
| **Home-based Pulmonary Rehabilitation (HPR)** | • Individual self-initiated home-based PR program with motivational and professional counseling, supported by health technology.<br>• The first session is a home visit from an experienced respiratory physiotherapist/nurse; to establish exercise goals and formal exercise prescription; provide a mindfulness program and instruction in use of a home diary and assess inhaler technique; assess exercise safety in the home environment.<br>• Receives 15 minutes of motivational counselling and program adjustment weekly by phone or online meeting, choice of patient preference. Consistent with the principles of motivational interviewing, asking the patient if and why he/she might want to increase the exercise program (desire); how this might be done in case of acceptance (ability); what would be the most important benefits in doing more exercise (reasons); and how important it is for the patient to do more exercise at this time (need).<br>• The overall goal for the patients in general is to achieve at least thirty minutes of self-initiated muscle-endurance based exercise three days a week.<br>• The prescribed physical exercises and mindfulness programs are available on the tablet as an instructed guidance.<br>• Education materials are delivered in paperback together with tablet and exercise equipment but also available as audio-book chapter on tablet in layman language. |

| | |
|---|---|
| | • Additionally, daily physical activity of at least thirty minutes by modalities accessible to the patient, e.g. in- or outdoor walking activities of in bouts of minimum 10 minutes. At the initial visit the physiotherapist/nurse accompanies the participant on an initial walk consistent with the patient's abilities (e.g. ability to walk stairs/hills).<br>• In a home diary the patients are instructed to record when, where and how often they do exercise and mindfulness; what might get in the way of their plans this week; and what can be altered.<br>• Exercise equipment is a package of a step-box and pairs of dumbbells weight from 1 to 5 kilo (heavier dumbbells available on request). |
| **Control group** | • Patients randomized in the control group will receive usual care; medication, contact and scheduled controls for the first 10 weeks. After 10 weeks, controls will be offered to participate in the maintenance exercise program for 15-month (65-weeks) free of charge or to continue as control without maintenance. The 10-, 35- and 75-weeks follow-up from baseline is the same as the intervention groups. If a patient changes their mind and wish to participate in a conventional hospital- og community-based PR program, it will be granted since this is a high recommended treatment (e.g. rehabilitation after hospital admitted exacerbation). |
| **Maintenance program** | • During the 15-month maintenance program, patients are asked to continue at home with a similar program to that completed in the intervention twice weekly with tablet and exercise equipment available and they are additionally offered group supervised exercise, mindfulness and dialogue/consultation (physiotherapist/nurse) once weekly for 60 minutes throughout the 15-month maintenance period. The overall goal for the patient is to achieve sustainable exercise habits and maintain or improve gains from the intervention. |

| **Exercise content** | | | |
|---|---|---|---|
| **Homed-based pulmonary rehabilitation (video-assisted)** | | **Pulmonary tele-rehabilitation (Supervised)** | |
| **Exercise type** | **Intensity and progression** | **Exercise type** | **Intensity and progression** |
| **Warm-up**<br>- Heel uprisings<br>- Knee extension<br>- Rear deltoid row<br>- Chest press movement<br>- Vertical shoulder press<br>- Walking various<br>- Leg curl<br>- Leg swing<br>- Squats<br>**(duration 5-10min)** | Non-specific intensity<br><br>Purpose:<br>- increase body temperature<br>- cardiorespiratory warm-up<br>- muscle and tendon warm-up | Warm-up<br>- Heel uprisings<br>- Knee extension<br>- Rear deltoid row<br>- Chest press movement<br>- Vertical shoulder press<br>- Walking on site<br>- Side to side walking<br>- Leg curl<br>- Leg swing<br>- Squats<br>**(duration 5min)** | Non-specific intensity<br><br>Purpose:<br>- increase body temperature<br>- cardiorespiratory warm-up<br>- muscle and tendon warm-up |
| **Exercises**<br>Test of choice:<br>1. Sit-to-stand from chair<br>2. Biceps curl – shoulder press<br>3. Step-up on step-box<br>4. Bent over rowing<br>5. Sit-to-stand from chair<br>6. Front raise dumbbells<br>**(duration 25min)** | *Intensity*<br>BORG CR-10 dyspnea 4-7<br>OR<br>*20-40 seconds ~ 8-25RM*<br>*4 sets of each exercise*<br>*rest between sets 40-20sec*<br>*Adjustment / Progression*<br>Weekly program/load/design adjustment after consultation and motivational interview with PT/RN | ***high repetitive/time-based muscle-endurance training***<br><br>Performed in numeric order:<br>1. Sit-to-stand from chair<br>2. Biceps curl – shoulderpress<br>3. Step-up on step-box<br>4. Bent over rowing<br>5. Sit-to-stand from chair<br>6. Front raise dumbbells<br>**(duration 25min)** | *Intensity*<br>*20-40 seconds ~ 8-25RM*<br>*4 sets of each exercise*<br>*rest between sets 40-20sec*<br>*Load: body weight + 1 to 20kg external weight*<br>*Progression*<br>Familiarization<br>week 1-2, work of 20sec/ rest 40sec<br>Progression 1<br>week 3-6, work of 30sec/ rest 30sec<br>Progression 2<br>week 7-10, work of 40sec/ rest 20sec<br>Progression with external weights individually adjusted |
| **Mindfulness**<br>**at least 15min/week)**<br><br>Program of choice<br>-Awareness of heartbeat and bloodflow<br>-Awareness of the body<br>-Awareness of sounds and thoughts<br>-Mindfull stretch | *Adjustment / Progression*<br>Week adjustment after consultation/motivational with PT/RN<br><br>Talk around mediating effects | **Mindfulness**<br>**25min every 2nd session**<br><br>Program of choice<br>-Awareness of heartbeat and bloodflow<br>-Awareness of the body<br>-Awareness of sounds and thoughts<br>-Mindfull stretch | *Adjustment / Progression*<br>Talk around mediating effects |
| **Cool-down**<br>- Breathing exercise<br>- Pursed lip breathe<br>- Relaxation exercise<br>- Yoga exercises<br>**(duration 5-10min)** | Non-specific intensity, progression or choice of exercise | ***Individual non-specific rest***<br>- Breathing/relax exercises<br>- Pursed lip breathe<br>- Resting on a chair<br>- Use of rest-room<br>- Intake water/ fruit/ other<br>**(duration 5min)** | Non-specific intensity, progression or choice of exercise |
| Health professional responsible: Physiotherapist<br>Monitoring of intensity may vary. Hospitals are expected to use either objective (pulse or Watt monitoring) or subjective (CR Borg scale for dyspnoea) intensity monitoring.<br>Resistance training, evaluated for progression by counting of maximal repetition and estimate of a new optional weight/resistance within 8-25 repetitions | | Health profession responsible: Physiotherapist<br>Monitoring (CR Borg scale for dyspnea 4-7) and rests needed due to dyspnea or muscle fatigue.<br>Patient counted repetitions every 6th session.<br>Excess >25 repetitions an increase in external weight load. | |

| Education content | | | |
|---|---|---|---|
| **Homed-based pulmonary rehabilitation (video-assisted)** | | **Pulmonary tele-rehabilitation** | |
| **Topic/themes** | **Communication/ learning form** | **Topic/themes** | **Communication/ learning form** |
| - COPD and treatment<br>- Importance of smoking cessation<br>- Importance of daily physical activity and exercise<br>- Importance of nutrition<br>- Medication and use of devices<br>- Early signs of exacerbation and action plan<br>- Use of nebulizer apparatus and oxygen apparatus<br>**(duration 60 min once a week)** | Topics/themes of patient choice discussed during phone call:<br>- Information<br>- Dialogue<br>- Reflection exercises<br>- Practical exercise<br>- Focus increasing individual self-competences<br>- network and sharing experiences | - COPD and treatment<br>- Importance of smoking cessation<br>- Importance of daily physical activity and exercise<br>- Importance of nutrition<br>- Medication and use of devices<br>- Early signs of exacerbation and action plan<br>- Use of nebulizer apparatus and oxygen apparatus<br>- Repetition and group needs<br>**(duration 3 x 20 min/week)**<br>Every second session is mindfulness exercises<br>-Awareness of heartbeat and bloodflow<br>-Awareness of the body<br>-Awareness of sounds and thoughts<br>-Mindfull stretch | Topics/themes promoted as a combination of:<br>- Information<br>- Dialogue<br>- Reflection exercises<br>- Practical exercise<br>- Focus on increasing individual self-competences<br>- network and sharing experiences |
| Self-initiated by patient and phone discussions weekly with HCP (RN/PT) | | Health professional responsible: Respiratory nurse | |

## Appendices 2

***Fysisk præstations målinger:***

1. *30sec- sit-to-stand-test (30STS) (Jones CJ et. 1999)* ***(standard test i Lunge-rehabilitering)***

*30-sec sit-to-stand test (30sec-STS)* er en indirekte måling af muskelstyrke af benene.
Testen udføres fra en stol med ryglæn og en sædehøjde svarende til 45-47 cm. Deltageren blive instrueret I at rejse og sætte sig så mange gang som muligt på 30 sekunder med armene krydset over brystet. Antal gennemførte rejse og sætte sig bliver registeret. Scoren "nul repetitioner" skrives hvis en patient ikke kan gennemføre testen (dvs. rejse-sætte-sig fra en stol uden brug af arme/hænder). Testen gentages to gange for at indfange evt. læringseffekt. Der holdes en pause på 5-minutter imellem de to forsøg. Det bedste testresultat anvendes. Testen anvendes som ***standard test i Lunge-rehabilitering og i ambulant medicinsk behandling.***

2. *Udholdenhed* – 6 min walking test (6MWT) (ERS/ATS technical guideline 2014) ***(standard test i Lunge-rehabilitering) OBS! Den test gennemføres kun på de patenter som vælger at få udført undersøgelse på hospitalerne.***

*6-minute walk test (6MWT*) anvendes til måling gang udholdenhed og gang kapacitet. Testen er anses som GOLD standard gang test I lungemedicin og anvendes som effektmål i lungerehabilitering og den danske regioners kvalitetsdatabase (RKKP-DRKOL). Gangtesten gennemføres på en 20 meter lang gangbane. Patient instrueres i at gå så langt som muligt på 6-minutter, og modtager undervejs standardiserede opmuntring fraser. Testen gennemføres to gange med 30 minutters pause imellem for at eliminere læringseffekt. Det bedste testresultat anvendes. Testen anvendes som ***standard test i Lunge-rehabilitering og i ambulant medicinsk behandling.***

3. *Håndgrebs styrke over-ekstremitet* - Jamar dynamometer (Robert HC et al. 2011)

The Jamar dynamometer (Preston, MI, USA) er et justerbart håndtag som kan måle håndgrebsstyrke. Håndgrebsstørrelsen kan justeres fra 2.5 til 7.5cm. Denne type håndgrebs-dynamometer udbredt i rehabilitering men er ikke en standard anvendt test. Procedure gennemføres siddende med albue bøjet i 90 grade og underarmen støttende på et armlæn. Patienten bliver instrueret til at klemme så hårdt som muligt om håndgrebet i 3-5 sekunder. Proceduren gennemføres tre gange med hver hånd med 10-20sekunders pause imellem hvert håndgrebsforsøg.

4. *Fysisk funktion* – five-time- sit-to-stand-test (5T-STS) (Jones SE et. 2014)

*Tiden på at rejse-sætte-sig* er et inddirekte måling af eksplosiv muskelkraft af benene.
Testen udføres fra en stol med ryglæn og en sædehøjde svarende til 45-47 cm. Deltageren blive instrueret I at rejse og sætte sig 5-gange så hurtigt som muligt med armene krydset over brystet.
Tiden brugt på 5- rejse-sætte-sig registres. Testen gentages to gange for at indfange evt. lærringseffekt. Der holdes en pause på 5-minutter imellem de to forsøg. Det bedste test-resultat anvendes.
Scoren "nul repetitioner" skrives hvis en patient ikke kan gennemføre testen (dvs. rejse-sætte-sig fra en stol uden brug af arme/hænder)

5. *Statisk og dynamisk balance* - (stående balance og 3-meter gang test) (Mequita R et al. 2016 and Guralnik JM etal. 1994)

a) *Stående balance testes ud fra tre forskellige positioner (Statisk balance)*:
- Stående med samlede fødder
- Stående i semi-tandemstand (forreste fods hæl ved siden af bagerste fods storetå)
- Stående i tandemstand (forreste fods hæl foran bagerste fods storetå)

Instruktioner:
Patient skal være i stand til at stå uden brug af krykstok eller rollator-
*"Jeg vil gerne teste din stående balance i forskellige stillinger, jeg viser dig nu hvilke og hvordan"*.
*"Lad mig understrege at du kun skal kun udføre stillingen hvis du føler dig tryg ved det"*.
"Føler du dig tryg?" og "har du nogen spørgsmål?"

Den sundhedsprofessionelle stå ved siden af patienten når testen gennemføres.

Tiden er afgørende under scoring af testen og hver position skal maksimalt holdes i 10 sekunder. Et sekund svarer til et point. Der kan scores mellem 0-30 point. Testpersonens samlede score udgøres af summen af de trin, der bliver gennemført.
- En score på 10 point, viser at personen fuldførte første position med samlede fødder og der fortsættes til semi-tandemstand.
- En score på 20 point viser, at personen fuldførte positionen med samlede fødder, og deslige fuldførte anden position semi-tandemstand. Kun hvis personen kan stå i semi-tandemstand i 10 sekunder gennemføres sidste position, tandemstand.
- En score på 30 viser, at testpersonen kan stå i alle tre positioner i 10 sekunder.

b) *3-meter gang test (dynamisk balance)*
Testen vurderer den intuitive normale gang hastighed hos patienten. Der anvendes gangredskab hvis det anvendes under dagliggang.

***Instruktion:***
*1. Dette er gang distancen på 3 meter du skal (viser af sundhedsprofessionel). Du skal nu gå fra denne afmærkning til (vises) til den anden ende 3 meter fremme. Du skal gå I dit vanlige gangtempo, ligesom hvis du gik udenfor på gaden*

*2. Den Sundhedsprofessionelle viser hvordan.*

*3. "Går hele vejen forbi markeringen 3 meter fremme før du stopper. Jeg går lige bagved dig. Føler du dig tryg ved testen?*

*4. Hav patienten stående med fødderne ved startmarkering.*

*5. Du skal starter efter kommandoen parat-gå.*

*6. Tiden på stopuret starter når patienten begynder at gå*

*7. Den Sundhedsprofessionelle går bagved patienten*

*8. Tiden stoppes når fødderne krydser slutmarkering 3 meter fremme.*

***Selvrapporteret målinger:***

Patients modtager en kort instruktion om skemaerne og udfylder dernæst spørgeskemaerne I et uforstyrret rum uden involvering af den sundhedsprofessionelle person.

*1.Selvrapporteret luftvejssymptomer* - COPD Assessment Test (CAT) ***(standard test i Lunge-rehabilitering)***

*COPD Assessment Test (CAT)* består af otte spørgsmål der omhandler patient-oplevede respirations symptomer nu og her. Hvert spørgsmål scores fra 0-5 (0 indikere ingen symptomer, 5 værst tænkelige symptomer) der summeres til en samlet score fra 0-40 point. Skemaet er ***standard skema i lunge-rehabilitering og i ambulant medicinsk behandling.***

*2. Selvrapporteret angst og depression symptomer* - Hospital Anxiety and Depression Scale (HADS) ***(standard skema i Lunge-rehabilitering)***

*Hospital Anxiety and Depression Scale (HADS)* består af 14 spørgsmål omhandlende dagligdags situationer som giver anledning til bekymring, angst og nedtrykthed. Spørgeskemaet består af to subscore angst-symptomer (syv spørgsmål) og depressions symptomer (syv spørgsmål). Hvert spørgsmål scores fra 0 to 3 (0 = no symptoms). Skemaet er ikke et diagnostisk redskab.

*3. Selvrapporteret livkvalitet* – Euro-Qol (EQ-5D-3L)

Europ-Qol er et standardiseret spørgeskema. Skemaet anvendes i flertallet af danske befolkningsundersøgelser til at kvantificere den helbredsrelaterede livskvalitet og funktionsevne ud fra en subjektive vurdering. Det således muligt både at vurdere effekten af en behandling på krops- og aktivitetsniveau samt sammenligne med baggrundsbefolkningens generelle helbredstilstand.

4. *Selvrapporteret muskel og skelet smerter* – Brief Pain Inventory (BPI)
Brief Pain Inventory registrerer patientens oplevede smerter indenfor den seneste uge. Der spørges til smerte intensitet, lokalisation af smerter i kroppen, effekt af smertelindrende medicin (0-100%), samt smerte geners indvirkning på daglige aktiviteter, humør, evne til at gå, passe arbejde, forhold til sociale relationer.

5. *Selvrapporteret træthed og udmattelses symptomer* – Multidimensional Fatigue Inventory (MFI-20)
MFI-20 afdækker fem dimensioner af træthed: Generel træthed (General fatigue), Fysisk træthed (fysisk fatigue), Mental træthed (Mental fatigue), Nedsat motivation (Reduceret motivation) og Nedsat aktivitet (Reduceret aktivitet). Der stilles 20 spørgsmål ex. " Jeg føler mig træt", og patienten bedes om at indikerer i hvilken grad udsagnet er korrekt på en fem-punkts-skala fra (ja, det er rigtigt - Nej, det er ikke rigtigt).

Spørgeskemaet har til hensigt at måle træthed som en tilstand, følsom over for midlertidige ændringer, og patienten besvarer spørgeskemaet på baggrund af hvordan de har haft det i den senere tid.
