# Supplementary material for: Supervised pulmonary tele-rehabilitation and individualized home-based pulmonary rehabilitation for patients with COPD, unable to participate in center-based programs. The protocol for a multicenter randomized controlled trial - the REPORT study
Source: PLoS One. 2025 Jan 7;20(1):e0312742. doi: 10.1371/journal.pone.0312742 (PMC11706455; doi:10.1371/journal.pone.0312742)
Supplement: S1 File — (PDF) [file pone.0312742.s003.pdf]

REPORT study – translated the 16th of May 2024
Journal number: P-2022-245-13101, 25.05.2022

TRANSLATION OF APPROVAL OF TRIAL FROM the Danish Data Protection Agency, Denmark. (Translated from Danish to English)

Page 1 – is blank in the approval (standard from the Danish Data Protection Agency).

The Capital Region of Denmark - Research Law

Report
Registration of research project

This serves as documentation for registration in Region Hovedstaden - Research Law's internal list of active research projects.

Project name: Rethink Pulmonary Rehabilitation – a randomized, multicenter intervention study

Approval number: P-2022-245-13101

Group companies: Capital Region of Denmark

REPORT study – translated the 16th of May 2024
Journal number: P-2022-245-13101, 25.05.2022

Description: Background for the project
The common disease chronic obstructive pulmonary disease (COPD) is related to age and smoking and costs approximately 8.5 billion DKK annually in citizens/patients' contact with and treatment in the healthcare system. It is estimated that 12% of all Danish adults over 45 have COPD, of which 40,000 people have severe disease. Improved treatment options and increasing life expectancy mean that the number of people with rehabilitation-requiring COPD is increasing and placing increased demands on optimal resource use in the healthcare system, also in the years to come.

International and national authorities and scientific societies emphasize that pulmonary rehabilitation is a central cornerstone in standard treatment of COPD on par with smoking cessation and medical treatment. There is robust documentation that pulmonary rehabilitation improves disease symptoms and quality of life and reduces healthcare costs. Despite the compelling documentation, less than 30% of all people with COPD receive pulmonary rehabilitation. Distance to rehabilitation centers in municipalities and hospitals is particularly challenging, especially for the most vulnerable and those with severe symptomatic disease. There are currently no alternative evidence-based offers or methods to the established pulmonary rehabilitation at the municipal health centers and hospitals.

There is a fundamental need to rethink and establish new evidence-based rehabilitation models and retention efforts. The potential and evidence for individualized home-based pulmonary rehabilitation (HLR) and group-supervised pulmonary telerehabilitation (PTR) is in demand globally and nationally.

Purpose of the project:
The randomized trial aims to investigate the short-term effect of 10 weeks of structured individual home-based pulmonary rehabilitation (HLR) and 10 weeks of supervised group-based pulmonary telerehabilitation (PTR) versus usual care (usual medical treatment and control visits) in people with moderate to very severe COPD who decline offers of pulmonary rehabilitation in hospital or municipality.

Subsequently, the maintaining effect (15 months) of a weekly supervised group-based pulmonary telerehabilitation session versus usual care over a period of 65 weeks is investigated.

Method:
The study includes data collection on: socio-demographics, anthropometry, physical function (endurance, physical activity, muscle strength, balance), self-reported symptoms of anxiety, depression, pain, sleep, respiratory symptoms, quality of life, and patient statements.
Data is collected at weeks 0, 10, 35, and 75. From week 0-10, the intervention is delivered, and from week 11-75, maintenance efforts are provided.

Design:
The study is a three-arm multicenter randomized controlled trial (RCT), with a subsequent 15-month maintenance intervention involving pulmonary outpatient clinics in the Capital Region of Denmark.

Inclusion:
As part of the annual outpatient COPD control and personal conversation at the affiliated hospital, with the general practitioner, or in a health center, all COPD patients should be assessed for pulmonary rehabilitation needs according to national recommendations. If pulmonary rehabilitation is deemed clinically justified, the patient is usually offered pulmonary rehabilitation. COPD patients who do not wish to or do not feel capable of participating in pulmonary rehabilitation may be offered to participate in the project "Rethink Pulmonary Rehabilitation - a randomized, multicenter intervention study" if they meet the participation criteria.

If the clinical staff (nurses and doctors) assess that the patient meets the criteria, the patient is invited to participate in the project by the clinical staff. If the patient agrees to be contacted by the trial coordinator, contact regarding potential participation will occur 3-7 days after the trial coordinator has received consent and contact information from the clinical staff.

The oral information session is offered to take place in an undisturbed room at the hospital, over the phone, or in the patient's own home depending on the individual patient's preference. Patients are encouraged to bring a family

member, friend, or acquaintance with them.

At the oral information meeting, it is explained that the project is based on voluntary participation, and that the project participant always has the opportunity to contact the trial supervisor or project leader for further information, as well as the right to withdraw from the project at any time and without justification. Furthermore, the participant is informed about the project, its benefits, drawbacks, risks, and the participant's rights.

Participants are informed about the extent of commitment required for the studies. Additionally, it is emphasized that all confidential and private information disclosed during the project is subject to confidentiality.

If the patient decides to participate in the trial, they are asked to sign a consent form. It is highlighted that the patient has the right to a consideration period before signing the consent form. The patient is requested to make a final decision and sign the consent form within 24 hours. The deadline for final consent can be extended if necessary.

Inclusion Criteria/Exclusion Criteria:

You can participate in the project if you:

- Are 40 years or older
- Have a diagnosis of COPD with moderate to very severe airway obstruction, medically defined as FEV1/FVC <70% (diagnosis) and FEV1 <80%

And meet the criteria below:
1. Unable to transport yourself and/or participate in conventional hospital- or community-based lung rehabilitation when offered through the lung outpatient clinic, your own doctor, or health center
2. A healthcare professional assesses that you have severe respiratory symptoms and/or frequent exacerbations equivalent to GOLD group B, C, or D
3. Can rise from and sit down on a chair (chair height 44-46cm) and walk 10 meters without assistance (with/without a walking aid e.g., a cane)
4. Can lift both arms horizontally with at least a 1-kilogram weight in each hand

Unfortunately, you cannot participate if you:
- Have participated in conventional (hospital or community-based) lung rehabilitation within the last 24 months.
- Have another serious illness where the doctor deems the test or exercise program unsuitable for you
- Have memory issues that make it difficult to understand basic information and instructions
- Have hearing and/or vision impairments that prevent you from following instructions and information
- Do not understand and/or speak Danish

Hypothesis:
Interventions HLR and PTR are expected to be equivalent to each other and superior to standard treatment in terms of respiratory symptoms as the primary outcome.

Expected Benefits of the Project:
The project is assumed to demonstrate a better effect of individualized home-based lung rehabilitation (HLR) and group-supervised lung tele-rehabilitation (LTR) on quality of life, anxiety and depression, activity level, and physical function compared to "standard treatment" in individuals with moderate to very severe COPD. The results of the project are expected to contribute to future recommendations and new evidence-based delivery methods of lung rehabilitation for individuals with moderate to very severe COPD.

Purpose: Health science research

Number of registered participants: 185

REPORT study – translated the 16th of May 2024
Journal number: P-2022-245-13101, 25.05.2022

Approval date: 20-05-2022

End date of data processing: 04-12-2030

Data sources:
Data processors: Statistics Denmark - research machine, Danish Health Data Authority - research machine

Sources: Region H - Retrieval of medical records

Danish Health Data Authority, National Patient Register, Cause of Death Register, Rehabilitation Services

Primary responsible
Henrik Hansen, trial supervisor, Postdoc, PhD, Physiotherapist,
Pulmonary Research Unit/Pulmonary Department,
Hvidovre Hospital
Phone: 2894 6780
Email: henrik.hansen.09@regionh.dk
Pulmonary Research Unit, center 2, Hvidovre Hospital,
Kettegård alle 30, 2650 Hvidovre

Contact person
Henrik Hansen, trial supervisor, Postdoc, PhD, Physiotherapist,
Pulmonary Research Unit/Pulmonary Department,
Hvidovre Hospital
Phone: 2894 6780
Email: henrik.hansen.09@regionh.dk
Pulmonary Research Unit, center 2, Hvidovre Hospital,
Kettegård alle 30, 2650 Hvidovre
