# Supplementary material for: Supervised pulmonary tele-rehabilitation and individualized home-based pulmonary rehabilitation for patients with COPD, unable to participate in center-based programs. The protocol for a multicenter randomized controlled trial - the REPORT study
Source: PLoS One. 2025 Jan 7;20(1):e0312742. doi: 10.1371/journal.pone.0312742 (PMC11706455; doi:10.1371/journal.pone.0312742)
Supplement: Attachment — Submitted filename: Response to Reviewers.docx [file pone.0312742.s004.docx]

**Response to Reviewers**

Hvidovre Hospital, 8^th^ of August 2024

Dear Academic Editor and Reviewers

Thank you for reviewing our manuscript and for your comments. We are very glad for your comments as it will improve our manuscript.

**Concerning journal requirements from Academic Editor:**

Ad. 1. The manuscript has been checked to meet the PLOS ONE’s style requirements (main body and title, authors affiliations).

Ad. 2. The funders had no role in design etc., this in now stated in the revised cover letter and in the article (please see line 40-41).

Ad. 3. Dataset.

The authors are not able to provide our datasets for public use due to ethical laws and very strict juristic data protection law in Denmark. Any possible access or sharing demands a part application to; (1) Danish Data Protection Agency, (2) Ethics Committee of the Capital Region, (3) National Health Data Authorities. Only if the applications are approved data will be considered available for sharing. The authors will not be able to support this process and a prolonged process must be expected.

Ad. 4. Ethics statement have been moved to the Method section and deleted in other section (please see line 285-293).

Ad. 5. Separate captions for each figure are now included in our manuscript.

Ad. 6. The reference list has been reviewed. I have found some discrepancies, a duplicate of same article reference. This have been corrected. The reference list does not contain any references to retracted articles.

**Concerning Reviewers comments:**

Ad. 1. No revisions made.

Ad. 2. No revisions made.

Ad. 3. No revisions made.

Ad. 4. Concerning data policy.

Data access in Denmark are under very strict juristic data protection law. Any possible access or sharing demands a part application to; (1) Danish Data Protection Agency, (2) Ethics Committee of the Capital Region, (3) National Health Data Authorities. Only if the applications are approved data will be considered available for sharing. The authors will not be able to support this process and a prolonged process must be expected.

Ad. 5. No revisions made.

Ad. 6.

\# Reviewer 1:

- 6.1: Change have been made (please see line 643)
- 6.2: Change have been made by our statistician (please see line 652)
- 6.3.: Change have been made (please see line 659)
- 6.4.: Change have been made (please see line 847)
- 6.5.: Extra information have been provided in the manuscript (please see line 851-856)
- 6.7.: Change have been made (please see line 880)

#Reviewer 2:

- 6.1.: We do not assess cognitive impairment with any standardized tests in our clinical practice and in other studies conducted at our research department. As described on page 10, line 241 in the manuscript, the clinical impression/assessment is based on the judgment of an outpatient respiratory nurse and a respiratory physician who refer the patient to the research study. Additionally, in cases of doubt concerning patient eligibility, such as cognitive impairment or comorbid conditions, the project's responsible respiratory physician and principal investigator are consulted for advice, and a pre-meeting is planned, after which the final decision is made. Finally, if the patient is unable to understand and follow the instructions and surveys during the baseline/inclusion assessment, the blinded assessor will contact the study's responsible PI for a prompt decision on whether to proceed or exclude the patient, with reasons provided.
  We have specified this process in more detail in the section “Eligibility Criteria.” We hope this clarifies your question. Please see page 10, lines 243-248.
- 6.2.: Concerning PRT home-based procedure and location of PTR – this is now more clearly stated in the manuscript.

Concerning the differences of the two groups – PTR and HPR – an extra short and concise table have been uploaded in the manuscript. Thank you for your recommendation.

- 6.3.: Concerning reasons for resignation and/or declining PR, this information is registered in a study database for further use in the final assessment. This information is now added in the manuscript (please see line 243-246).

With regards for randomization and severity of functional impairment, the statistical analyses will

include adjustment for FEV1, among others. Unfortunately, we are not able to obtain data on DLCO

and/or hyperinflation as this is not a standard examination in Danish outpatient clinics unless a pulmonologist deems it necessary. It is not possible for the investigators to change the randomization method as recruitment has begun and are ongoing. Furthermore, FEV1 is only used as a descriptive value and not an effect value.

In our author group we have discussed that our section concerning blinding needed more details. We have therefore added information, please see line 319-326. Furthermore, I have found a type error (in the section concerning 1-min STS test) and I have corrected it.

I hope that the abovementioned is satisfactory otherwise please do not hesitate to contact us again and thank you for reviewing our manuscript.

With best regards

Christina Nielsen
